# Perceived non-smoking norms and motivation to stop smoking, quit attempts, and cessation: a cross-sectional study in England

**DOI:** 10.1038/s41598-020-67003-8

**Published:** 2020-06-26

**Authors:** Sarah E. Jackson, Hannah Proudfoot, Jamie Brown, Katherine East, Sara C. Hitchman, Lion Shahab

**Affiliations:** 1 Department of Behavioural Science and Health, University College London, London, UK; 2 National Addiction Centre, Institute of Psychiatry, Psychology and Neuroscience, King’s College London, London, UK; 3 Shaping Public Health Policies To Reduce Inequalities and Harm (SPECTRUM) Consortium, Edinburgh, UK

**Keywords:** Public health, Epidemiology

## Abstract

This study examined the prevalence of non-smoking norms in England and their associations with motivation to stop smoking, quit attempts, and cessation. Data were from a representative cross-sectional survey of 1,521 adults (301 combustible tobacco smokers). Descriptive non-smoking norms were endorsed, with just 16% of adults (12% of smokers) believing smoking was uncommon. Injunctive non-smoking norms were more prevalent, with 60–77% of adults (17–48% of smokers) viewing smoking as something of which others disapproved. Personal non-smoking norms were also prevalent among all adults (73% indicated they would prefer to live with a non-smoker) but not smokers (69% had no preference). Smokers who endorsed stronger descriptive non-smoking norms had increased odds of reporting high motivation to stop smoking (OR_adj_ = 1.63, 95%CI 1.06–2.52). Female (but not male) past-year smokers who endorsed stronger injunctive (OR_adj_ = 2.19, 95%CI 1.41–3.42) and personal (OR_adj_ = 1.90, 95%CI 1.29–2.82) non-smoking norms had increased odds of having made a past-year quit attempt. In conclusion, perceived descriptive non-smoking norms are not held by the majority of adults in England. Injunctive and personal non-smoking norms are prevalent among all adults but lower among smokers. There is some evidence that smokers – in particular, women – who endorse stronger non-smoking norms are more likely to be motivated to stop smoking and to make a quit attempt.

## Introduction

Reducing tobacco smoking prevalence remains a public health priority^1^. Social norms are an important influence on human behaviour^2^ and may be affected by interventions or policy to promote smoking cessation^3^. For example, in England, smoke-free legislation was implemented in 2007 banning tobacco smoking in enclosed public places and workplaces; communicating a strong non-smoking message^4^. Smoking prevalence has since fallen by over a third; from 24% in 2007 to 14% in 2018^5^. Norms can be conveyed not only through the behaviour of others (i.e. descriptive norms) but also through the perceived acceptability of the behaviour by others (i.e. injunctive norms) or one’s own attitudes and values (i.e. personal norms)^6^. Understanding these smoking norms and their associations with quitting activity is important for the design of future smoking cessation interventions and tobacco control policies.

The different types of social norms may influence smoking behaviour in different ways, leading some smokers to quit and others not. In terms of descriptive norms, there is strong evidence that smoking is a socially contagious behaviour: people are substantially more likely to smoke if others in their social networks (e.g. friends, family, colleagues) smoke^7^. Importantly, groups of interconnected people appear to quit in concert. Knowing someone who has stopped smoking increases the likelihood of a quit attempt^7^. In terms of injunctive norms, at a population-level, smoke-free legislation and smoking cessation campaigns convey that smoking is less acceptable and such initiatives have been associated with a reduction in smoking prevalence^8–12^. These policies and interventions may gradually lead to non-smoking norms in society^13^ which may be perceived as social pressure to stop smoking^14^ and may subsequently trigger quit attempts^15–17^. In terms of personal norms, internalisation of descriptive and injunctive norms may lead to self-reflection and changes in personal attitudes towards smoking; for example, perception of others’ disapproval of smoking may reduce one’s own perception of smoking as acceptable^18^. However, non-smoking norms clearly do not prompt all smokers to quit^19^. Instead, other motivations may prove more influential or conflicting local norms may prevail, such as whether one’s partner or close friends smoke^20^. Behavioural norms are often inconsistent in this manner as they are communicated by many different sources^21^.

Norms may also affect individuals differently based on various sociodemographic factors. For instance, people may respond differently to non-smoking norms due to gender socialisation. Subjective quitting norms (significant others’ expectations) have been found to be significantly and positively associated with quitting intentions in female smokers but not in male smokers^20^. This could be the result of greater social disapproval towards female smoking compared with male smoking^20^, particularly in countries where smoking prevalence is much higher in men than women (e.g. China, Malaysia, the Middle East^22,23^). In England, the gender gap is less pronounced, but men are more likely than women to smoke (16.4% vs. 12.6%)^5^. Research suggests that normative influences also have diverse effects in different cultures which may impact smokers’ quitting behaviour considerably^24^. In addition, policies that discourage smoking and emphasise its unacceptability could alienate more socioeconomically deprived smokers who have fewer resources available to help them quit^25,26^. Smoking-related factors such as level of cigarette addiction may also influence the effect of social norms on quitting behaviour, with smokers who are more addicted being possibly less likely to be influenced by norms^27^. Understanding the extent to which associations between non-smoking norms and quitting behaviour are moderated by sociodemographic and smoking characteristics is important for understanding whether policies and interventions might exacerbate social inequalities in smoking and health. It could also help understand differences in responses to interventions and inform how norms might best be used to increase the effectiveness of a message.

This study aimed to investigate the extent to which non-smoking norms are associated with different tobacco smoking cessation activities^28^, including motivation to quit, quit attempts, and cessation itself, and whether these associations are moderated by sociodemographic factors. Specifically, we aimed to address the following research questions:

1. To what extent do adults in England, and tobacco smokers specifically, endorse descriptive, injunctive, and personal non-smoking norms?
2. Among current smokers, to what extent are descriptive, injunctive, and personal non-smoking norms associated with motivation to stop smoking, adjusting for relevant confounding variables?
3. Among past-year smokers, to what extent are descriptive, injunctive, and personal non-smoking norms associated with (i) having made at least one serious quit attempt in the past year and (ii) cessation, adjusting for relevant confounding variables?
4. To what extent are associations between non-smoking norms and quitting activity moderated by age, sex, social grade, and level of cigarette addiction?

## Method

### Design

Data were used from the Smoking Toolkit Study, an ongoing monthly cross-sectional survey of a representative sample of the general population of adults in England designed to provide insights into population-wide influences on tobacco smoking and cessation by monitoring trends on a range of variables relating to smoking^29^. Data are collected by market research company Ipsos MORI. Each month, a new sample of approximately 1,700 adults aged ≥16 years is selected using a form of random location sampling (of whom ~400 are tobacco smokers). Participants complete a face‐to‐face computer‐assisted survey with a trained interviewer. Estimates of sociodemographic characteristics and smoking prevalence have been shown to be nationally representative based on comparisons with other national surveys and recorded sales^29,30^.

The present study used data from respondents to the survey in March 2016, as items on non-smoking norms were introduced in this wave as a one-time measurement, alongside the usual measures that assess current smoking status, motivation to quit smoking, past quit attempts, and other smoking characteristics.

### Population

A total of 1,689 adults were surveyed in March 2016. We used data from all respondents for descriptive analyses of non-smoking norms, and from respondents who reported smoking combustible tobacco daily or occasionally during the past year (‘past-year smokers’) for analyses of the associations between non-smoking norms and quitting activity.

### Measures

#### Explanatory variables

Explanatory variables included two items assessing descriptive non-smoking norms, four items assessing injunctive non-smoking norms, and one item assessing personal norms/attitudes towards smoking. The development of these measures was based on desk reviews of current measures, cognitive testing, and pilot testing, with experts and other stakeholders consulted at each stage (for full details see^31^).

Descriptive interpersonal norms were assessed with a measure of the number of close personal connections who smoke: “*Think of the five people you feel most close to. These could be your partner, family members, friends, colleagues or acquaintances. Thinking of these FIVE people, how many of them, if any, are tobacco cigarette smokers?*” Response options were 0, 1, 2, 3, 4, 5, or don’t know.

Descriptive societal norms were assessed with a measure of the commonality of smoking: “*Do you think that smoking tobacco cigarettes is… (very uncommon, uncommon, neither common nor uncommon, common, very common, don’t know)?”*

Injunctive interpersonal norms towards smoking were assessed with measures of partner, family, and friends’ approval of smoking: “*How do (you think) each of the following people (would) feel about you smoking tobacco cigarettes? (i) Your partner/spouse, (ii) Your immediate family, (iii) Your close friends?”* Response options were: strongly disapprove, disapprove, neither approve nor disapprove, approve, strongly approve, not applicable, or don’t know.

Injunctive societal norms towards smoking were assessed with a measure of perceived public approval of smoking: “*In your opinion, do people in general approve or disapprove of people smoking tobacco cigarettes?”* Response options were: strongly disapprove, disapprove, neither approve nor disapprove, approve, strongly approve, or don’t know.

Personal norms towards smoking were assessed with the question: “*Please imagine that you need to find a new lodger or housemate. Would you…? (only live with a non-smoker, prefer a non-smoker but consider a smoker, have no preference between smokers and non-smokers, prefer a smoker but consider a non-smoker, only live with smoker, don’t know)*.”

For each of these items, responses were coded as continuous variables, from 0 to 5 for descriptive interpersonal norms and 0 to 4 for all other norms. Scores of 0 indicated strong pro-smoking norms (i.e. having 5 close acquaintances who smoke, thinking smoking is very common, thinking people strongly approve of smoking, or choosing only to live with a smoker). Scores of 4 (or 5 for number of close acquaintances who smoke) indicated strong non-smoking norms (i.e. having 0 close acquaintances who smoke, thinking smoking is very uncommon, thinking people strongly disapprove of smoking, or choosing only to live with a non-smoker). ‘Don’t know’ responses were excluded.

Scores on the two items assessing descriptive norms were averaged and four items assessing injunctive norms were averaged to create composite measures for analysis alongside the single-item measure of personal norms. For analyses of associations with our outcomes of interest, z-scores were used to facilitate comparison across each of the three different types of norms.

#### Outcome variables

Outcome variables were motivation to stop smoking, quit attempts, and cessation.

Motivation to stop smoking among current smokers was assessed with the Motivation to Stop Scale^32^. This single-item measure asks: “*Which of the following best describes you? (1) I REALLY want to stop smoking and intend to in the next month; (2) I REALLY want to stop smoking and intend to in the next 3 months; (3) I want to stop smoking and hope to soon; (4) I REALLY want to stop smoking but I don’t know when I will; (5) I want to stop smoking but haven’t thought about when; (6) I think I should stop smoking but don’t really want to; (7) I don’t want to stop smoking*.” We dichotomised responses to distinguish between those who reported high motivation to stop smoking in the next three months (responses 1 or 2) and those with low motivation (all other response options)^33,34^.

Quit attempts were assessed by asking past-year smokers: “*How many serious attempts to stop smoking have you made in the last 12 months? By serious attempt I mean you decided that you would try to make sure you never smoked again. Please include any attempt that you are currently making, and please include any successful attempt within the last 12 months*.” Those who reported at least one quit attempt were coded 1 and those who reported no quit attempts in the last 12 months were coded 0.

Cessation was analysed as the proportion of past-year smokers who reported abstinence at the time of the survey.

#### Covariates

Sociodemographic covariates included in our analyses were age, sex, ethnicity, and socioeconomic position. Ethnicity was categorised as white vs. non-white. Socioeconomic position was assessed using the National Readership Survey’s occupational social grade index and categorised for analysis as ABC1 (managerial, administrative and professional occupations) vs. C2DE (semi‐routine and routine occupations, manual occupations, never workers and long‐term unemployed)^35^.

Smoking-related covariates varied by outcome, selected *a priori* in accordance with existing evidence^36,37^. Analyses of quit attempts just controlled for sociodemographic variables listed above. Analyses of motivation to stop  smoking controlled for sociodemographic variables and the number of past-year quit attempts (categorised as 0, 1, ≥1). Analyses of cessation controlled for sociodemographic variables, the number of past-year quit attempts, and strength of urges to smoke (an indicator of level of cigarette addiction that closely predicts relapse in this population^38^).

### Statistical analysis

Analyses were conducted in SPSS v.24 on complete cases. The analysis plan was pre-registered on Open Science Framework (https://osf.io/dxqza/).

The distribution of responses to items assessing non-smoking norms for the whole sample and by smoking status (non-smoker, past-year smoker [including both current smokers and those who quit in the past year], current smoker) were summarised using descriptive statistics. The reason we report data for both past-year smokers and current smokers, despite the considerable overlap of these samples, is to facilitate interpretation of subsequent analyses of associations with motivation to stop smoking (which was assessed in current smokers) and quit attempts and cessation (which were assessed in past-year smokers). To provide nationally representative estimates, descriptive data were weighted using rim (marginal) weighting to match the English population profile relevant to the time the survey was conducted on dimensions of age, social grade, region, tenure, ethnicity, and working status within sex. We used t-tests (continuous variables) and chi-square tests (categorical variables) to compare non-smokers with past-year smokers and current smokers.

Associations of descriptive, injunctive, and personal non-smoking norms (as z-scores) with motivation to stop smoking, quit attempts, and cessation were analysed using multivariable logistic regression, with and without adjustment for the above-mentioned covariates.

In order to test for moderation of associations by sociodemographic characteristics, we replicated these models adding two-way interactions between non-smoking norms (z-scores) and (i) age, (ii) sex, (iii) social grade, and (iv) level of addiction (measured by strength of urges to smoke). We had also pre-specified that we would test interactions with ethnicity, but this was not feasible given the sample of smokers was predominantly white (see Table 1). Where there was evidence of moderation, we ran stratified analyses to provide more information as to the nature of the differences between groups.

**Table 1:** Sample characteristics.

	Whole sample (*n* = 1,521)	Non-smokers (1) (*n* = 1,216)	Past-year smokers^a^ (2) (*n* = 301)	Current smokers (3) (*n* = 275)	*p* (2) vs. (1)	*p* (3) vs. (1)
Age in years, % (*n*)					<0.001	<0.001
16–24	14.6 (222)	13.2 (160)	19.8 (59)	19.2 (53)	—	—
25–34	16.9 (257)	15.5 (188)	22.8 (68)	22.9 (63)	—	—
35–44	16.8 (256)	16.2 (197)	19.0 (57)	19.5 (54)	—	—
45–54	17.2 (262)	16.6 (202)	20.1 (60)	20.2 (56)	—	—
55–64	14.2 (216)	14.9 (181)	11.5 (35)	11.5 (32)	—	—
≥65	20.3 (308)	23.7 (288)	6.9 (21)	6.7 (18)	—	—
Female sex, % (*n*)	49.6 (754)	51.2 (622)	48.0 (144)	48.6 (134)	0.238	0.320
White ethnicity, % (*n*)	86.7 (1319)	85.5 (1040)	91.4 (275)	91.7 (253)	0.006	0.005
Social grade C2DE, % (*n*)	45.1 (685)	41.6 (506)	58.2 (175)	58.8 (162)	<0.001	<0.001
Smoking status, % (*n*)						
Current smoker	18.6 (283)	—	92.7 (279)	100 (275)	—	—
Recent (≤12 months) ex-smoker	1.4 (22)	—	7.3 (22)	—	—	—
Long-term (>12 months) ex-smoker	18.3 (279)	22.9 (279)	—	—	—	—
Never smoker	61.6 (937)	77.1 (937)	—	—	—	—
Number of past-year quit attempts, % (*n*)						
0	—	—	72.8 (219)	77.3 (213)	—	—
1	—	—	19.2 (58)	14.9 (41)	—	—
≥2	—	—	8.0 (24)	7.8 (21)	—	—
Strength of urges (0–5), mean (SD)	—	—	1.92 (1.14)	2.02 (1.09)	—	—
High motivation to quit, % (*n*)	—	—	11.7 (32)^b^	11.7 (32)	—	—
Attempted to quit in past 12 months, % (*n*)	—	—	27.2 (82)	22.7 (62)	—	—
Smoking cessation in past 12 months, % (*n*)	—	—	8.4 (25)	—	—	—

Note: Weighted data.

^a^Includes current smokers and those who quit in the past year. *N* = 2 were excluded because of missing data on past-year quit attempts; thus, the number of non-smokers and past-year smokers does not sum exactly to the number of participants in the whole sample.

^b^Valid percentage shown, only relevant to current smokers.

In an unplanned sensitivity analysis, we repeated our logistic regression models and moderation analyses separately for each of the two items making up the descriptive non-smoking norm measure (number of close personal connections who smoke and commonality of smoking), because responses to these variables were polarised in opposite directions (see results section).

In order to aid interpretation of non-significant associations, we calculated Bayes factors to differentiate between evidence for no effect from data insensitivity. Bayes factors were calculated using an online calculator (http://www.lifesci.sussex.ac.uk/home/Zoltan_Dienes/inference/Bayes.htm) with alternative hypotheses represented by half-normal distributions and the expected effect size set to OR = 2 on the basis of previous research into non-smoking norms and cessation behaviours^18^. Bayes factors ≥3 can be interpreted as evidence for the alternative hypothesis (and against the null), ≤1/3 as evidence for the null hypothesis, and between 1/3 and 3 suggest the data are insensitive to distinguish the alternative hypothesis from the null^39,40^.

### Ethics approval and consent to participate

Ethical approval for the STS was granted originally by the UCL Ethics Committee (ID 0498/001). The data are not collected by UCL but by an external market research company (Ipsos MORI) and are anonymized when received by UCL. All participants provided fully informed consent. The study was carried out in accordance with the Declaration of Helsinki.

## Results

A total of 1,689 adults aged ≥16 years responded to the survey. Complete data on the variables of interest were provided by 89.8% of respondents (weighted *n* = 1,521), of whom 301 (19.8%) were past-year smokers and 275 (18.1% of the total sample) were current smokers. The majority (73.4%) of missing cases were excluded by ‘don’t know’ responses to one or more of the non-smoking norm items. Descriptive characteristics of the whole sample, past-year smokers, and current smokers are shown in Table 1.

### Descriptive non-smoking norms

Descriptive data summarising the strength of non-smoking norms are shown in Table 2. In the whole sample of adults, most respondents (39.8%) reported that none of their five closest social connections were smokers but just 15.9% believed smoking was uncommon or very uncommon. Fewer past-year and current smokers than non-smokers reported that none of their closest social connections were smokers (14.9% and 13.8% compared with 45.9%, respectively). More than 10% of smokers reported that all five of their closest social connections were smokers; more than double than was reported by the whole sample. Despite this, there were no notable differences in the witnessed commonality of smoking, with similar minorities perceiving smoking to be uncommon or very uncommon to those observed in the whole sample.

**Table 2:** Strength of non-smoking norms held by the general population of adults, past-year smokers, and current smokers in England.

	Whole sample (*n* = 1,516)	Non-smokers (1) (*n* = 1,206)	Past-year smokers^a^ (2) (*n* = 308)	Current smokers (3) (*n* = 281)	*p* (2) vs. (1)	*p* (3) vs. (1)
**Descriptive norms**						
Number of close personal connections who smoke, % (*n*)					<0.001	<0.001
0. 5 – all of them	4.6 (70)	2.9 (35)	11.6 (35)	12.4 (34)	—	—
1. 4	5.5 (83)	3.5 (43)	12.5 (38)	12.9 (35)	—	—
2. 3	12.6 (191)	9.8 (119)	24.2 (73)	25.0 (69)	—	—
3. 2	15.6 (238)	14.9 (181)	18.8 (57)	18.1 (50)	—	—
4. 1	21.9 (334)	23.0 (280)	18.0 (54)	17.8 (49)	—	—
5. 0 – none of them	39.8 (605)	45.9 (559)	14.9 (45)	13.8 (38)	—	—
Witnessed commonality of smoking, % (*n*)					0.171	0.151
0. Very common	17.1 (261)	17.1 (208)	17.5 (53)	16.1 (44)	—	—
1. Common	49.7 (756)	49.7 (604)	50.0 (150)	50.1 (138)	—	—
2. Neither common nor uncommon	17.3 (264)	16.4 (200)	20.2 (61)	21.2 (58)	—	—
3. Uncommon	14.4 (219)	15.2 (185)	11.3 (34)	11.6 (32)	—	—
4. Very uncommon	1.5 (22)	1.6 (20)	0.9 (3)	0.9 (3)	—	—
Composite score (0–4.5)^b^, mean (SD)	2.49 (0.94)	2.62 (0.89)	1.96 (0.97)	1.94 (0.98)	<0.001	<0.001
**Injunctive norms**						
Partner’s approval^c^, % (*n*)					<0.001	<0.001
0. Strongly approve	0.8 (11)	0.5 (6)	1.9 (5)	2.1 (5)	—	—
1. Approve	7.0 (92)	4.6 (49)	17.1 (43)	17.8 (41)	—	—
2. Neither approve nor disapprove	18.2 (241)	12.0 (129)	43.1 (108)	43.1 (98)	—	—
3. Disapprove	30.3 (403)	31.8 (341)	24.5 (61)	24.8 (56)	—	—
4. Strongly disapprove	43.8 (581)	51.0 (548)	13.3 (33)	12.2 (28)	—	—
Family’s approval, % (*n*)					<0.001	<0.001
0. Strongly approve	0.7 (10)	0.3 (4)	2.2 (6)	2.3 (6)	—	—
1. Approve	5.0 (77)	3.5 (43)	11.3 (34)	12.2 (33)	—	—
2. Neither approve nor disapprove	17.7 (269)	12.2 (148)	38.8 (117)	41.1 (113)	—	—
3. Disapprove	33.9 (516)	34.1 (415)	33.6 (101)	32.0 (88)	—	—
4. Strongly disapprove	42.7 (649)	49.9 (607)	14.1 (42)	12.4 (34)	—	—
Friends’ approval, % (*n*)					<0.001	<0.001
0. Strongly approve	0.8 (12)	0.6 (7)	1.8 (5)	1.9 (5)	—	—
1. Approve	7.5 (114)	4.9 (59)	18.3 (55)	18.6 (51)	—	—
2. Neither approve nor disapprove	31.5 (479)	23.5 (286)	62.8 (189)	64.4 (177)	—	—
3. Disapprove	31.4 (477)	36.1 (439)	12.5 (37)	11.2 (31)	—	—
4. Strongly disapprove	28.8 (438)	34.9 (425)	4.7 (14)	3.9 (11)	—	—
Approval of people in general, % (*n*)					<0.001	<0.001
0. Strongly approve	0.8 (12)	0.6 (7)	1.6 (5)	1.8 (5)	—	—
1. Approve	7.2 (110)	6.5 (79)	10.3 (31)	10.3 (28)	—	—
2. Neither approve nor disapprove	23.6 (359)	20.0 (244)	36.9 (111)	37.7 (104)	—	—
3. Disapprove	54.2 (825)	57.3 (697)	42.7 (128)	42.3 (116)	—	—
4. Strongly disapprove	14.2 (215)	15.6 (190)	8.4 (25)	8.0 (22)	—	—
Composite score (0–4)^b^, mean (SD)	2.93 (0.75)	3.09 (0.69)	2.31 (0.67)	2.27 (0.66)	<0.001	<0.001
**Personal norms**						
Would you…, % (*n*)					<0.001	<0.001
0. Only live with a smoker	1.4 (21)	1.3 (16)	1.7 (5)	1.9 (5)		
1. Prefer smoker consider non-smoker	1.9 (29)	1.3 (16)	4.5 (13)	4.9 (13)	—	—
2. No preference between smokers and non-smokers	23.8 (362)	12.5 (152)	68.7 (206)	70.3 (194)	—	—
3. Prefer non-smoker consider smoker	15.7 (239)	16.3 (198)	13.6 (41)	12.4 (34)	—	—
4. Only live with a non-smoker	57.1 (869)	68.6 (835)	11.5 (35)	10.5 (29)	—	—
Score (0–4)^b^, mean (SD)	3.25 (0.97)	3.50 (0.86)	2.29 (0.79)	2.25 (0.78)	<0.001	<0.001

Note: Weighted data.

^a^Includes current smokers and those who quit in the past year. *N* = 2 were excluded because of missing data on past-year quit attempts; thus, the number of non-smokers and past-year smokers does not sum exactly to the number of participants in the whole sample.

^b^Higher scores indicate stronger non-smoking norms. The range of possible scores is given in parentheses.

^c^Valid percentages shown, since some respondents did not have a partner (*n* = 206 in whole sample).

### Injunctive non-smoking norms

In the whole sample, respondents largely perceived smoking to be something other people disapproved of: 74.1%, 76.6%, and 60.2% thought their partner, family, and close friends, respectively, did or would disapprove (or strongly disapprove) of them smoking. They held similar perceptions of societal attitudes towards smoking, with 68.4% of respondents indicating that they thought people in general disapproved of other people smoking. The proportion of smokers who thought their social connections disapproved (or strongly disapproved) of their smoking was comparatively lower than was observed in non-smokers: of past-year smokers, 37.8% said that their partner disapproved, 47.7% said their family disapproved, and just 17.2% said their friends disapproved. Around half (50.1%) said they thought people in general disapproved of other people smoking. Results were similar for current smokers.

### Personal non-smoking norms

In the whole sample, more than half (57.1%) of respondents said that they would only live with a non-smoker and an additional 15.7% said they would prefer a non-smoker. One in four (23.8%) had no preference, and <4% said they would prefer to live with a smoker. Smokers less commonly reported a preference for living with a non-smoker (25.1% of past-year smokers, 22.9% of current smokers), with most past-year smokers (68.7%) and current smokers (70.3%) indicating that they had no preference between smokers and non-smokers. Non-smokers reported a strong preference for living with a non-smoker, with 68.6% saying they would only live with a non-smoker and a further 16.3% preferring a non-smoker.

### Associations with quitting activity

Associations between non-smoking norms and quitting activity are shown in Table 3. After adjustment for covariates, current smokers who endorsed stronger descriptive non-smoking norms had significantly increased odds of reporting high motivation to stop smoking. However, past-year smokers were not significantly more (or less) likely to have made a quit attempt or to have quit smoking in the last 12 months, with data providing moderate evidence for the null hypothesis (Table S1). Similar results were observed when the components of the descriptive non-smoking norms measure (number of close personal connections who smoke and witnessed commonality of smoking) were analysed separately. The only notable exception was that smokers who perceived smoking to be more common were significantly less likely to have quit smoking in the last 12 months.

**Table 3:** Associations of non-smoking norm scores with motivation to stop smoking, quit attempts, and cessation.

	Motivation to stop smoking^a^		Quit attempts^b^		Cessation^b^	
	OR [95% CI] *p*	OR_adj_^c^ [95% CI] *p*	OR [95% CI] *p*	OR_adj_^d^ [95% CI] *p*	OR [95% CI] *p*	OR_adj_^e^ [95% CI] *p*
Descriptive norms	1.43 [1.00–2.04] 0.054	1.63 [1.06–2.52] 0.027	0.89 [0.69–1.15] 0.371	0.90 [0.69–1.18] 0.438	1.09 [0.73–1.61] 0.683	0.91 [0.53–1.55] 0.718
Number of close personal connections who smoke	1.16 [0.80–1.67] 0.429	1.46 [0.94–2.28] 0.092	0.86 [0.67–1.10] 0.230	0.85 [0.65–1.11] 0.222	1.34 [0.89–2.02] 0.156	1.28 [0.73–2.23] 0.394
Witnessed commonality of smoking	1.64 [1.16–2.33] 0.006	1.50 [1.00–2.26] 0.051	1.02 [0.79–1.31] 0.897	1.06 [0.81–1.39] 0.655	0.71 [0.46–1.10] 0.124	0.50 [0.26–0.93] 0.029
Injunctive norms	1.27 [0.88–1.85] 0.207	1.05 [0.67–1.64] 0.835	1.37 [1.06–1.79] 0.018	1.42 [1.08–1.88] 0.013	1.70 [1.12–2.57] 0.012	1.71 [0.92–3.19] 0.092
Personal norms	1.34 [0.94–1.89] 0.102	1.12 [0.74–1.70] 0.597	1.42 [1.11–1.83] 0.006	1.57 [1.20–2.06] 0.001	1.67 [1.17–2.39] 0.005	1.40 [0.82–2.39] 0.224

Note: Unweighted data. Explanatory variables (non-smoking norms) were analysed as z-scores, with higher scores indicating stronger non-smoking norms.

OR, odds ratio. 95% CI, 95% confidence interval.

^a^Current smokers.

^b^Past-year smokers.

^c^Adjusted for age, sex, ethnicity, social grade, and number of past-year quit attempts.

^d^Adjusted for age, sex, ethnicity, and social grade.

^e^Adjusted for age, sex, ethnicity, social grade, number of past-year quit attempts, and level of addiction (strength of urges to smoke).

Past-year smokers who endorsed stronger injunctive or personal non-smoking norms had significantly increased odds of having made a quit attempt in the last 12 months. However, there were no significant associations between injunctive or personal non-smoking norms and smoking cessation among current smokers, and data were insensitive to distinguish between a modest effect and no effect for motivation to stop smoking (Table S1).

### Moderation effects

There were no significant interactions between non-smoking norms and age, social grade, or (for cessation) level of addiction with quitting activity (Table S2). However, there was some evidence of moderation of associations by sex. While non-smoking norms appeared to be associated with motivation to stop smoking to a similar degree in men and women, descriptive, injunctive, and personal norms were more strongly associated with quit attempts (OR = 1.75, 95% CI 1.03–2.99, *p* = 0.040; OR = 2.24, 95% CI 1.26–4.00, *p* = 0.006; and OR = 1.73, 95% CI 1.01–2.96, *p* = 0.046, respectively), and descriptive norms (specifically, the number of close personal connections who smoke) were more strongly associated with cessation (OR = 3.43, 95% CI 1.04–11.33, *p* = 0.043), in women than men. Stratified analyses (Table 4) revealed that associations between stronger injunctive and personal non-smoking norms were significantly associated with increased odds of quit attempts in women but not in men. There was a significant *negative* association between strength of descriptive non-smoking norms and quit attempts in men but no significant association in women, with data proving insensitive (Table S1). Descriptive non-smoking norms were associated with reduced odds of cessation in men and increased odds of cessation in women. Associations did not reach statistical significance when analysed as a composite score: the data provided strong evidence for the null hypothesis (i.e. stronger non-smoking norms were not associated with increased cessation) for men but were insensitive for women (Table S1). However, when number of close personal connections who smoke was analysed separately from commonality of smoking, there was a significant association with increased odds of cessation in women (Table 4).

**Table 4:** Stratified analyses of significant interactions between non-smoking norms and sex.

Explanatory variable	Outcome	Men OR_adj_ [95% CI] *p*	Women OR_adj_ [95% CI] *p*
Descriptive norms	Quit attempts^a^	0.64 [0.42–0.98] 0.038	1.16 [0.80–1.69] 0.442
Injunctive norms	Quit attempts^a^	0.91 [0.60–1.39] 0.661	2.19 [1.41–3.42] 0.001
Personal norms	Quit attempts^a^	1.18 [0.78–1.77] 0.433	1.90 [1.29–2.82] 0.001
Descriptive norms	Cessation^b^	0.50 [0.22–1.17] 0.111	1.99 [0.69–5.74] 0.205
Number of close personal connections who smoke	Cessation^b^	0.74 [0.34–1.63] 0.455	5.93 [1.10–31.86] 0.038

Note: Unweighted data. Explanatory variables (non-smoking norms) were analysed as z-scores.

OR, odds ratio. 95% CI, 95% confidence interval.

^a^Adjusted for age, sex, ethnicity, and social grade.

^b^Adjusted for age, sex, ethnicity, social grade, number of past-year quit attempts, and level of addiction (strength of urges to smoke).

## Discussion

Perceived descriptive non-smoking norms were not held by the majority of adults in England, with only 16% of adults (12% of smokers) believing tobacco smoking to be uncommon or very uncommon and less than half (40%) of adults and just 15% of smokers reporting that none of their five closest social connections smoked. In contrast, injunctive non-smoking norms were more prevalent, with 60–77% of adults (17–48% of smokers) viewing smoking as something of which partners, family, friends, and people in general disapproved. Personal non-smoking norms were also prevalent among adults in general (73% indicated that they would prefer to live with a non-smoker) but not for smokers specifically (69% had no preference between a smoker and non-smoker). After adjustment for covariates, smokers who endorsed stronger descriptive non-smoking norms had significantly increased odds of reporting high motivation to stop smoking. Female (but not male) past-year smokers who endorsed stronger injunctive and personal non-smoking norms had significantly increased odds of having made a quit attempt in the last 12 months. Data on cessation were largely insensitive, although female past-year smokers who reported that a greater number of their close personal connections were smokers had significantly increased odds of cessation.

Our findings are consistent with several previous studies conducted in other countries. For example, data from the International Tobacco Control Project revealed positive associations between injunctive non-smoking norms (disapproval of close personal connections and society in general) and quitting intentions of smokers in Malaysia, Thailand, Canada, the USA, the UK, and Australia^24^. Another cross-sectional survey of German smokers showed that a descriptive quitting norm (“most people who are important to me have quit smoking themselves”) was associated with greater intention to quit^20^. A prospective study of Australian smokers found that disapproval of friends and family, embarrassment about being a smoker, and living with a recent quitter were associated with quitting intention and engagement in smoking limiting behaviours^18^. In the latter study, embarrassment (but not the other norms) was also associated with increased likelihood of talking about quitting and making a quit attempt^18^. Our results build on this literature by analysing three different types of non-smoking norm (descriptive, injunctive, personal) in relation to three different measures in the quitting process (motivation, quit attempts, cessation), and testing for moderation by relevant sociodemographic and smoking characteristics. Taken together, these findings suggest that interventions or policies that change smoking norms might be useful in helping to reduce smoking prevalence^3^. We found that many people (even non-smokers) believe smoking is a common behaviour. Descriptive norms tend to be most strongly associated with behaviour but are difficult to change. Interventions providing accurate information about how common smoking is (e.g. what proportion of people in England smoke) might help to change the perceived prevalence of smoking, and hence possibly motivation to stop smoking.

There was some evidence of moderation of associations between non-smoking norms and quitting behaviour by sex, with significant associations with quit attempts only observed in women. The previous German study similarly observed that subjective quitting norms (significant others’ expectations) were associated with quitting intentions in female but not male smokers^20^, but a recent study of low-income smokers in Baltimore in the US found the opposite, with cessation norms associated with quit attempts in men but not women^41^. We did not find evidence of moderation by age, social grade, or level of addiction. This suggests that while policies and interventions based on normative information may be more effective in encouraging women to quit than men, they are likely to have a similar impact on quitting behaviour across smokers of all ages, socioeconomic circumstances, and with higher and lower levels of addiction. Why male smokers may be less susceptible to normative social influence requires further investigation but may reflect men being less likely than women to follow social norms in general^42,43^. From a cross-cultural perspective, strong non-smoking norms for women in certain cultures (e.g. China, Malaysia, the Middle East) have resulted in markedly different smoking prevalence between men and women^22,23^. In such countries, norm-based interventions may be less effective in promoting cessation because smoking is more common and accepted (indicating pro-smoking norms) for men than women, and most of the target population (being male) are less influenced by non-smoking norms.

Strengths of the study include the nationally-representative sample and assessment of a range of quitting-related outcomes. There were also several limitations. The items assessing non-smoking norms were only included in one wave of the Smoking Toolkit Study, limiting the sample size and, thus, reducing statistical power to detect associations. Moreover, a substantial number of participants (~10% of those eligible) were excluded due to responding ‘don’t know’ to the non-smoking norm items. Bayes factors calculated for non-significant results indicated that the data were insensitive in a number of instances, so we were unable to distinguish between a modest effect and no effect. Replication in a larger sample is required to draw firm conclusions. The cross-sectional study design precludes inferences on causality. It is likely that associations between non-smoking norms and smoking/quitting are reciprocal, with non-smoking norms leading smokers to want to quit, and quitting leading to changes in smokers’ non-smoking norms. The data were collected four years ago (2016) and norms may have changed over this period as smoking prevalence has continued to decline. The measure used to assess descriptive interpersonal norms (number of close associates/family members who smoke) did not assess the extent to which respondents identified with the people they felt most close to. It is therefore possible that an individual may score highly on this measure, by having a high number of close personal connections who smoke, but have low identification with these references and thus be relatively uninfluenced by them due to a low shared social identity or affiliation^44,45^. Cognitive testing indicated respondents appeared to understanding that the ‘personal norms’ measure reflected a personal preference. However, the measure may have been influenced by factors that restricted ability to live with a smoker – for example, personal health reasons (e.g. asthma) or a housing contract that only permitted non-smokers – rather than personal attitudes or values. It may also be subject to various interpretations – for example, an individual may not have a strong preference about living with a smoker if smoking took place outside of the shared home. As such, it has limitations as a measure of broad personal attitudes towards smoking. The items assessing non-smoking norms were not gender-specific (i.e. they referred to people in general, rather than men or women specifically), which may be a limitation given our findings that tobacco smoking norms may influence behaviour to different extents in women compared with men. The influence of gender-specific norms could be explored in future research. The fact that the vast majority of participants were white meant we were unable to explore interactions between non-smoking norms and ethnicity, which may be important given previous research showing smoking norms can have very different influences on quitting behaviour across different ethnicities and cultures^22^. This is another direction for future research to focus on. Finally, all data were self-reported, with no biochemical verification of smoking status. However, this is not generally considered to be as problematic in surveys as it is in clinical trials, where participants may feel more pressure to claim abstinence^46^, and we have recently shown a close alignment between cigarette consumption in England estimated by population survey and sales data^46^.

## Conclusions

In England, most adults endorse strong injunctive and personal non-smoking norms but the majority do not perceive descriptive non-smoking norms. Tobacco smokers tend to endorse non-smoking norms less strongly relative to the general population. There is some evidence that smokers – in particular, women – who endorse stronger non-smoking norms are more motivated to stop smoking and more likely to make a quit attempt. However, the strength of associations varies according to the type of non-smoking norm (descriptive, injunctive, personal) and quitting construct (motivation, quit attempts, cessation).

## Supplementary information

Supplementary information.

## Data Availability

Data are available upon reasonable request.
