# Supplementary material for: Perceived non-smoking norms and motivation to stop smoking, quit attempts, and cessation: a cross-sectional study in England
Source: Sci Rep. 2020 Jun 26;10:10487. doi: 10.1038/s41598-020-67003-8 (PMC7320183; doi:10.1038/s41598-020-67003-8)
Supplement: Supplementary file 1 — Supplementary information. [file 41598_2020_67003_MOESM1_ESM.docx]

| **Table S1.** Bayes factors for non-significant associations | | | | |  |
| --- | --- | --- | --- | --- | --- |
|  | | **Motivation to stop smoking** | **Quit attempts** | **Cessation** | |
| **Descriptive norms** | |  |  |  | |
|  | Whole sample | - | 0.10  Moderate evidence for H0 | 0.27  Moderate evidence for H0 | |
|  | Men | - | - | 0.08  Strong evidence for H0 | |
|  | Women | - | 0.56  Data are insensitive | 1.09  Data are insensitive | |
| Number of close personal connections who smoke | |  |  |  | |
|  | Whole sample | 1.23  Data are insensitive | 0.07  Strong evidence for H0 | 0.81  Data are insensitive | |
|  | Men | - | - | 0.25  Moderate evidence for H0 | |
| Witnessed commonality of smoking | |  |  |  | |
|  | Whole sample | 1.45  Data are insensitive | 0.30  Moderate evidence for H0 | - | |
| **Injunctive norms** | |  |  |  | |
|  | Whole sample | 0.39  Data are insensitive | - | 1.26  Data are insensitive | |
|  | Men | - | 0.20  Moderate evidence for H0 | - | |
| **Personal norms** | |  |  |  | |
|  | Whole sample | 0.48  Data are insensitive | - | 1.01  Data are insensitive | |
|  | Men | - | 0.61  Data are insensitive | - | |
| H0, null hypothesis (i.e. stronger non-smoking norms are not associated with increased odds of the outcome).  Bayes factors ≥3 can be interpreted as evidence for the alternative hypothesis (and against the null), ≤1/3 as evidence for the null hypothesis, and between 1/3 and 3 suggest the data are insensitive to distinguish the alternative hypothesis from the null. | | | | |  |

| **Table S2.** *p* values for tests of interactions between non-smoking norms and age, sex, social grade, and level of addiction | | | | | |
| --- | --- | --- | --- | --- | --- |
|  | | **Motivation to stop smoking** | **Quit attempts** | **Cessation** |  |
| **Descriptive norms** | |  |  |  |  |
|  | Age | 0.075 | 0.127 | 0.746 |  |
|  | Sex | 0.788 | **0.040** | **0.043** |  |
|  | Social grade | 0.302 | 0.676 | 0.327 |  |
|  | Level of addiction | - | - | 0.341 |  |
| Number of close social connections who smoke | |  |  |  |  |
|  | Age | 0.099 | 0.069 | 0.552 |  |
|  | Sex | 0.912 | 0.119 | **0.032** |  |
|  | Social grade | 0.194 | 0.586 | 0.527 |  |
|  | Level of addiction | - | - | 0.507 |  |
| Witness commonality of smoking | |  |  |  |  |
|  | Age | 0.328 | 0.323 | 0.379 |  |
|  | Sex | 0.835 | 0.080 | 0.170 |  |
|  | Social grade | 0.771 | 0.838 | 0.335 |  |
|  | Level of addiction | - | - | 0.337 |  |
| **Injunctive norms** | |  |  |  |  |
|  | Age | 0.238 | 0.317 | 0.253 |  |
|  | Sex | 0.978 | **0.006** | 0.772 |  |
|  | Social grade | 0.755 | 0.541 | 0.758 |  |
|  | Level of addiction | - | - | 0.803 |  |
| **Personal norms** | |  |  |  |  |
|  | Age | 0.461 | 0.645 | 0.786 |  |
|  | Sex | 0.349 | **0.046** | 0.538 |  |
|  | Social grade | 0.867 | 0.454 | 0.970 |  |
|  | Level of addiction | - | - | 0.302 |  |
| Note: Unweighted data. p values in bold font were statistically significant (<0.05). Only these interactions were investigated further (see Table 4). | | | | | |
